# Genome Sequences of Three Cruciviruses Found in the Willamette Valley (Oregon)

**DOI:** 10.1128/MRA.00447-19

**Published:** 2019-06-06

**Authors:** Ignacio de la Higuera, Ellis L. Torrance, Alyssa A. Pratt, George W. Kasun, Amberlee Maluenda, Kenneth M. Stedman

**Affiliations:** a Department of Biology, Portland State University, Portland, Oregon, USA; b Center for Life in Extreme Environments, Portland State University, Portland, Oregon, USA; DOE Joint Genome Institute

## Abstract

Cruciviruses are single-stranded DNA (ssDNA) viruses whose genomes suggest the possibility of gene transfer between DNA and RNA viruses. Many crucivirus genome sequences have been found in metagenomic data sets, although no crucivirus has been isolated. Here, we present the complete genome sequences of three cruciviruses recovered from environmental samples from Oregon.

## Abstract (precis)

Cruciviruses are single-stranded DNA (ssDNA) viruses whose genomes suggest the possibility of gene transfer between DNA and RNA viruses. Many crucivirus genome sequences have been found in metagenomic data sets, although no crucivirus has been isolated.

## ANNOUNCEMENT

First described as RNA-DNA hybrid or chimeric viruses (1, 2), cruciviruses are a group of viruses whose genomes are circular molecules of single-stranded DNA (ssDNA) that typically contain 2 open reading frames (ORFs). One ORF encodes a replication-associated protein (Rep), which is involved in the replication of single-stranded DNA virus genomes. The other ORF encodes a capsid protein that is homologous to capsid proteins of plant-infecting tombusviruses, a family of RNA viruses. The presence of two genes that are similar in viral groups with disparate genomic properties is of great interest from an evolutionary standpoint, as it implies gene transfer between unrelated groups of viruses.

Crucivirus genomes have been previously detected in different viromes spanning hot springs to peat soils (1–6), but no virus had been isolated to date, and their host range and ecology remain obscure. Thus, it is important to test for the presence of these viruses in a given environment as a first step toward their isolation and characterization.

**FIG 1:**
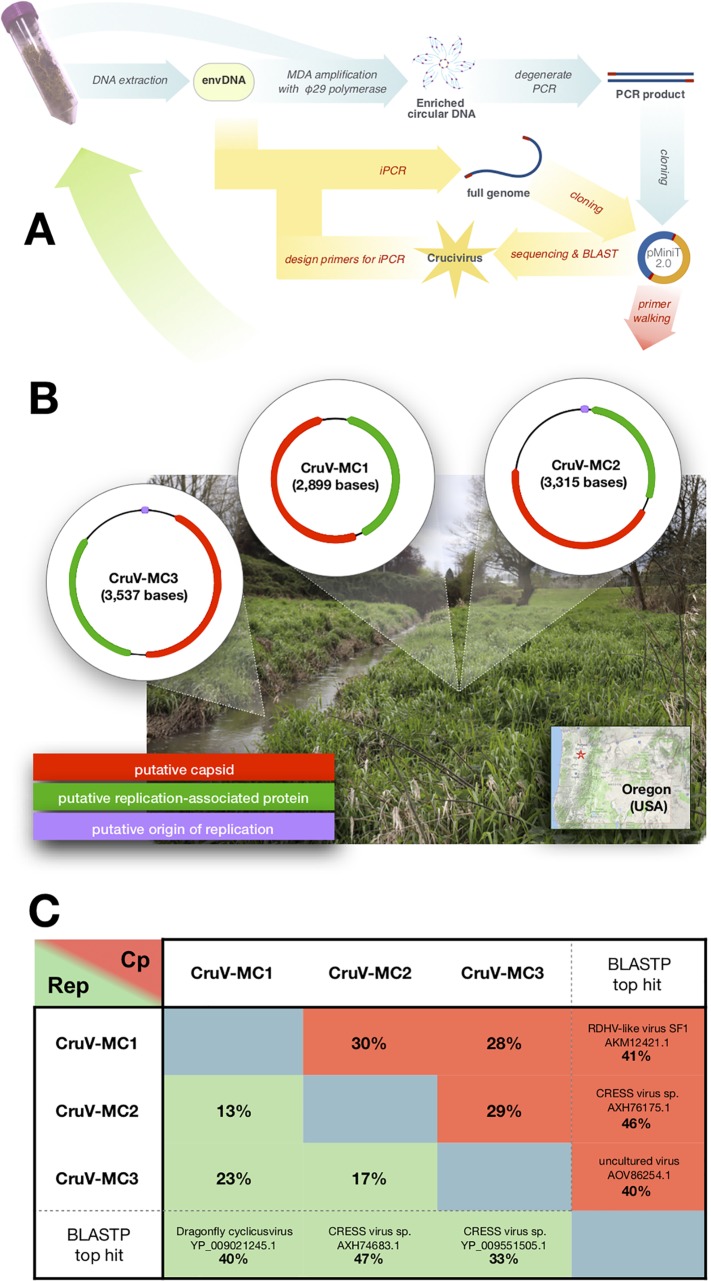
(A) Outline of methodology used for detection and recovery of cruciviral sequences from environmental samples. Blue arrows represent the first steps after sample collection, and yellow arrows are the follow-up steps. envDNA, environmental DNA; MDA, multiple displacement amplification; iPCR, inverse PCR. (B) Genome structure of the cruciviruses found in Mill Creek, OR. Putative capsid protein genes are depicted in green, putative Rep genes are in red, and putative origins of replication are in purple. A photograph of the sampled area is shown in the background. The location of Woodburn is indicated by a star on the map in the bottom-right corner. (C) Table indicating pairwise identity for the replication-associated protein (Rep) and the capsid protein (Cp) between each of the new cruciviral genomes. Sequences were aligned with MAFFT (L-INS-i; BLOSUM45; gap open penalty = 2.5; offset value = 0.123) in Geneious 11.0.4. The name, accession number, and percent identity of the best BLASTP hit for the new Cp and Rep sequences are indicated in the last column and row. Searches were performed on the NCBI Web server using the GenBank nonredundant protein sequence database on 29 April 2019. None of the top hits for Rep correspond to crucivirus sequences, while all of the Cp hits do.

*Mill Creek crucivirus 1* (CruV-MC1; 2,899 bases; 41% GC content), *Mill Creek crucivirus 2* (CruV-MC2; 3,315 bases; 40% GC content), and *Mill Creek crucivirus 3* (CruV-MC3; 3,537 bases; 37.2% GC content), the 3 cruciviral genomes presented here, were obtained from samples collected from, or adjacent to, Mill Creek (Fig. 1A and B), within the city limits of Woodburn, OR, located in the Willamette Valley (coordinates 45°09′14.1″N, 122°50′40.1″W).

The sequences of CruV-MC1 and CruV-MC2 were recovered from soil samples (∼10 g; pH, ∼5) collected on 29 March 2017. DNA was extracted using a Mo Bio PowerLyzer PowerSoil kit, following the manufacturer’s instructions. The environmental DNA was amplified with phi29 polymerase (NEB) using a degenerate primer matching a conserved region of crucivirus capsid genes, with the sequence 5′-RTNGARTG*Y*G-3′ (asterisks indicate 3′-phosphorothioation).

The CruV-MC3 sequence was found in water (∼500 ml; pH, ∼5) sampled on 13 August 2018, from the creek adjacent to the soil sample location. Undiluted water (10 μl) was directly amplified with phi29 DNA polymerase primed by random hexamers without previous DNA isolation (7).

Amplified DNA was precipitated with ethanol and sodium or ammonium acetate. The DNA was used as the template for a degenerate PCR (primers 5′-GGTWCWRTHATWATGKCTACTSAWTAYAA-3′ and 5′-KWAACCCAYAGYTCRCC-3′) targeting the conserved capsid domain. The amplicons were cloned into pMiniT 2.0 (NEB) and sequenced by dideoxy terminator sequencing (Eurofins MWG Operon). The sequences obtained were used to design specific primers to amplify the whole crucivirus genomes by inverse PCR with Phusion DNA polymerase (NEB). The PCR products were cloned into pMiniT 2.0 and sequenced by dideoxy terminator sequencing (Eurofins MWG Operon). A primer walking strategy with ∼700-base reads was used to sequence each genome on both strands to ensure a Phred quality score greater than 20 for the entirety of each genome. Completeness of the circular genomes was confirmed by amplification and sequencing of the gapped regions and/or by protein sequence alignment.

Genomes were annotated using Geneious 11.0.4 by predicting ORFs and manually analyzing the protein sequences with BLASTP (8). Whereas the capsid protein is relatively similar in the three genomes, the Rep protein of CruV-MC2 is less similar to those of CruV-MC1 and Cruv-MC3 (Fig. 1C). In addition to the ORFs, secondary structures in the DNA sequence that putatively serve as origins of replication (9) were predicted and annotated. CruV-MC2 and CruV-MC3 contain the conserved nonanucleotides TAGTATTAC and GAGTATTAC, respectively, within a stem-loop structure confirmed by mfold (10).

### Data availability.

The information and genomic sequences of CruV-MC1, CruV-MC2, and CruV-MC3 were deposited at DDBJ/ENA/GenBank under the accession numbers MK679543, MK679544, and MK679545, respectively.
